# Generalized Phase Tailoring of Arbitrary Orthogonal Polarizations in Meta‐Structure with High‐Order Geometric Symmetry

**DOI:** 10.1002/advs.202504918

**Published:** 2025-05-14

**Authors:** Kai Qu, Ke Chen, Qi Hu, Weixu Yang, Junming Zhao, Tian Jiang, Yijun Feng

**Affiliations:** ^1^ School of Electronic Science and Engineering Nanjing University Nanjing 210023 China; ^2^ Suzhou Laboratory Suzhou 215000 China

**Keywords:** generalized geometric phase, high symmetry, metasurface, polarization control, wavefront engineering

## Abstract

Symmetry, a core principle of aesthetics, plays a crucial role in both physics and mathematics. Recent investigations into high‐symmetry meta‐structures (C*m*, *m* ≥ 3) have revealed intriguing concepts and phenomena in optics and materials science. However, increasing symmetry introduces challenges in tailoring anisotropy, limiting the potential of highly symmetric structures for functional wavefront engineering. While nonlinear geometric Berry phase and generalized geometric phase have enabled circular polarization control in C*m* (*m* ≥ 3) meta‐structures, these approaches are inherently spin‐dependent and restricted to circular polarization states. Here, shape tailoring, including modifications to unit cell dimension and C*m* meta‐structure parameters is presented, to effectively enhance the anisotropy and phase control capabilities of C*m* (*m* ≥ 3) meta‐structures. By further incorporating generalized geometric phase, independent phase control of arbitrary orthogonal polarization states is achieved, and validated both numerically and experimentally. This generalized framework enhances the wavefront tailoring capacity of C*m* (*m* ≥ 3) meta‐structures and has substantial potential for integrating flexible wavefront functions with high‐symmetry‐driven exotic phenomena. Moreover, this approach offers inspiration for further applications in fields such as condensed matter physics and materials science, i.e., integrating the symmetry of the unit cell and lattice.

## Graphical abstract

A generalized methodology is proposed for C*m* (*m* ≥ 3) meta‐structures to achieve independent phase control of arbitrary orthogonal polarizations. By integrating orientation tuning with structural tailoring, this approach extends spin‐based designs to polarization‐diverse wavefront engineering, enabling multi‐functional and high‐efficiency meta‐devices, effectively enhancing the wavefront tailoring capability of high‐symmetry meta‐structures, and unlocking new opportunities in symmetry‐driven photonic applications.

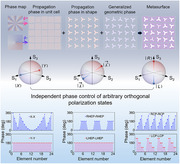

## 1 Introduction

Geometric symmetry has profound implications across many branches of physics, from condensed matter physics to quantum systems. In optics and photonics, sub‐wavelength structures with various symmetries, including axial and rotational symmetry, play critical roles in establishing foundational isotropic or anisotropic optical elements.^[^ ^1^ ^]^ Recently, metasurfaces have emerged as a fascinating platform for achieving peculiar electromagnetic phenomena and enabling diverse wavefront‐tailoring functionalities at sub‐wavelength scales.^[^ ^2^, ^3^, ^4^, ^5^, ^6^ ^]^ Meta‐atom with onefold or twofold rotational symmetry (denoted as C1 or C2) represents the two fundamental configurations of rotationally symmetric elements of metasurfaces.^[^ ^7^, ^8^ ^]^ Leveraging the Pancharatnam–Berry phase (PB phase, or named geometric phase),^[^ ^9^, ^10^ ^]^ which arises from the spin‐coupling effect,^[^ ^11^, ^12^ ^]^ PB phase meta‐structures with C1 and C2 symmetry have been widely used in various functional metasurfaces by spatially rotating each meta‐atom to achieve full phase coverage.^[^ ^7^, ^13^, ^14^, ^15^, ^16^, ^17^, ^18^ ^]^ Distinct from C1 and C2 meta‐structures, although C*m* meta‐structures with *m*‐fold rotational invariance (*m* ≥ 3) can provide new physics for disruptive applications and induce exotic phenomena in optics and photonics, such as implementing bound states in the continuum,^[^ ^19^ ^]^ harmonic generation,^[^ ^1^, ^20^, ^21^ ^]^ the rotational Doppler effect,^[^ ^22^, ^23^ ^]^ and topological state of matter,^[^ ^24^, ^25^, ^26^ ^]^ they remain less developed in wavefront engineering compared to the classical C1 and C2 configurations.

**Figure 1:**
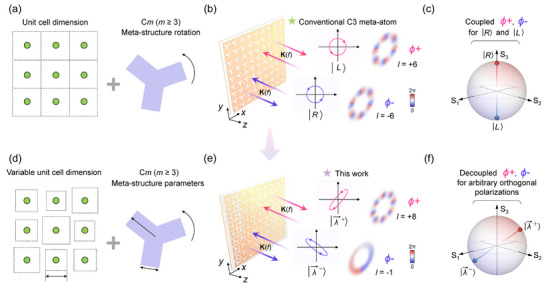
a) Conventional high‐order geometric phase strategy based on C*m* meta‐structures. b) Schematic diagram of generalized geometric phase (using C3 as an example). K(*f*) represents the wave factor at frequency *f*; |*L*〉 and |*R*〉 denote left‐handed and right‐handed circular polarization states, respectively. *ϕ* ^+^ and *ϕ* ^−^ represent phase shifts for a pair of orthogonal polarization states. *l* is the mode of generated orbit angular momentum. c) Locations of polarization states on the Poincaré sphere where the conventional approach is applicable, limited to the two poles of the sphere. In the Poincaré sphere, *S*_1_‐*S*_3_ are the three stokes parameters. d) The proposed strategy is based on variable unit cell dimension and meta‐structure parameters. e) Schematic diagram of the proposed metasurface, composed of high‐symmetry meta‐atoms, which is utilized to independently tailor the wavefront of arbitrary orthogonal polarization states. ${|\vec{\lambda }\rangle }^{\pm }$ are two arbitrary orthogonal polarization states. f) Locations of polarization states on the Poincaré sphere where the proposed approach is applicable, theoretically extending to any two symmetric points on the sphere.

The C*m* meta‐structures with high‐order symmetry are initially explored in nonlinear optics, allowing continuous control of nonlinear phase in harmonic waves. A meta‐structure with C*m* symmetry only allows the generation of *t* = *lm* ± 1 (where *l* is an integer) orders of harmonic waves ^[^ ^27^, ^28^ ^]^ and imposes phase factor e^(^ *^t^* ^−1)^ *^σθ^* or e^(^ *^t^* ^+1)^ *^σθ^* (*θ* is the rotation angle of meta‐structure and *σ* is the spin angular momentum) onto either the same or the opposite circular polarization state of the fundamental wave.^[^ ^29^ ^]^ However, phase responses between different spin states remain deeply correlated due to the fixed phase factor form, limiting their independent control for arbitrary phase targets. On the other hand, in linear optics, C*m* meta‐structures have traditionally been viewed as isotropic and unsuitable for constructing geometric phase meta‐atoms. In 2021, Xie et al. revealed that meta‐structures with high symmetry can also generate high‐order geometric phase, which depends on both the symmetry of meta‐structure and the arrangement lattice.^[^ ^30^ ^]^ Inspired by this, a novel global selection rule is proposed in nonlinear optics, jointly governed by the symmetries of both the meta‐atom and the lattice. This unified framework not only enables frequency conversions previously forbidden by traditional selection rules, but also reveals a more comprehensive relationship between the nonlinear PB phase and the meta‐atomic rotation angle, thereby paving the way for advanced nonlinear meta‐devices with potential applications in high‐harmonic wavefront shaping.^[^ ^7^, ^31^, ^32^, ^33^ ^]^ In addition, in linear optics the general geometric phase can be expansively written as *Φ* = ± 2*mθ* (when *n* is odd) or *Φ* = ± *mθ* (when *n* is even), allowing phase shift ratios relative to rotation angle variation to exceed ± 2, reaching values such as ± 6, ± 8, and beyond. As shown in **Figure** **1**a, the generalized phase is realized by deploying fixed‐size unit cells in a homogeneous lattice while rotating the C*m* meta‐structures within each unit cell. However, Most of C*m* meta‐structures under this strategy still suffer from spin coupling in wavefront tailoring for a pair of spin channels. For example, as illustrated in Figure 1b, a C3‐based spiral phase meta‐plate generates orbital angular momentum in opposite modes (± 6) because opposite phase shifts (*ϕ* ^+^ and *ϕ* ^−^) are always imposed on each circular polarization state. Moreover, a series of meta‐devices based on high‐symmetry meta‐structures have been developed, demonstrating excellent performance.^[^ ^34^, ^35^, ^36^, ^37^ ^]^ For example, combining propagation and generalized geometric phases enables asymmetric spin–orbit interaction with much higher efficiency than C2 meta‐atoms of the same period and height.^[^ ^37^ ^]^ However, current high‐order symmetry meta‐structures are still limited to circular polarization states, represented by the north and south poles on the Poincaré sphere, as depicted in Figure 1c. For more general cases involving linear and elliptical polarization pairs, represented by two points symmetrically located on opposite sides of the Poincaré sphere, C*m* meta‐structures have not yet been demonstrated with independent flexible wavefront tailoring capabilities. Overall, while higher symmetry in photonic structures has the potential to produce fascinating physical effects, it also presents significant challenges in basic photonic anisotropy tailoring and phase engineering.

Here, we propose a generalized methodology for meta‐structures with high‐order geometric symmetry C*m* (hereafter, unless otherwise specified, *m* ≥ 3) to achieve independent phase engineering for arbitrary orthogonal polarization states. As schematically shown in Figure 1d, by integrating orientation variation (generalized geometric phase) with the evolution of unit cell dimension (propagation phase with variable unit cell size) and C*m* meta‐structure parameters (propagation phase with variable high‐symmetry pattern), high‐symmetry meta‐structures can overcome the inherent limitation of spin coupling. This approach also allows for greater flexibility in tailoring meta‐structure anisotropy, effectively extending phase engineering beyond two spin channels to arbitrary orthogonal polarization states. To validate the capacity for flexible wavefront manipulation, we designed a series of polarization‐multiplexing meta‐devices composed of high‐order rotationally symmetric meta‐structures and verified experimentally at microwave frequency. Compared to the conventional geometric phase and generalized geometric phase, our strategy demonstrates the enhanced potential of C*m* meta‐structures in polarization‐diverse phase engineering. For example, the ability to achieve independent phase control (e.g., fully decoupled modes carrying orbital angular momentum) for arbitrary orthogonal polarization states (any two symmetric points on the Poincaré sphere) is illustrated in Figure 1e,f. This methodology paves the way for more flexible control of high‐symmetry meta‐structures and may facilitate the integration of exotic physical phenomena across multiple symmetries with versatile phase engineering into a single compact photonic device platform.

## 2 Results

### 2.1 Theoretical Analysis of Independent Phase Control via Cm (m ≥ 3) Meta‐Structures

We first delve into the theoretical analysis to demonstrate that highly rotationally symmetric meta‐structures can indeed enable arbitrary phase control for a pair of circularly polarized waves, or even any set of orthogonal polarization states.

To illustrate, we consider a reflection case in which independent wavefront tailoring of the incident orthogonal polarization states via a single C*m* meta‐atom can be expressed as 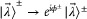, where 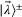 are two orthogonal polarization states and ϕ^±^ are the arbitrary phase profiles to be imposed on the two reflected light waves, respectively. By characterizing the polarization state in the form of the Jones vector, the two incident polarization states can be specifically written in the linear polarization basis as

(1)

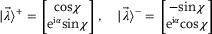

where *α* and *χ* set the polarization states. The reflected electric field can be calculated as 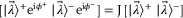, where ***J*** is the Jones matrix for this process and can be further written as

(2) $$J=e^{i\alpha }\left[\begin{array}{cc} e^{i\alpha }\left(e^{i\phi^{+}}\cos^{2}\chi +e^{i\phi^{-}}\sin^{2}\chi \right) & \frac{\sin\left(2\chi \right)}{2}\left(e^{i\phi^{+}}-e^{i\phi^{-}}\right)\\ \frac{\sin\left(2\chi \right)}{2}\left(e^{i\phi^{+}}-e^{i\phi^{-}}\right) & e^{-i\alpha }\left(e^{i\phi^{+}}\sin^{2}\chi +e^{i\phi^{-}}\cos^{2}\chi \right) \end{array}\right]$$

This matrix provides a general framework (further details about the derivation process are shown in Section S1 (Supporting Information). According to Ref. [38] achieving such phase manipulation requires the collaboration of the geometric phase (orientation variation of the meta‐atom) and propagation phase. For simplicity, we first identify the required rotation angle *θ* that satisfies the Jones matrix in Equation (2), however, the meta‐atom orientations cannot be intuitively observed from Equation (2). To derive the rotation angle according to the geometric phase embodied in the circular polarization components, we transform the Jones matrix in Equation (2) into a circular polarization basis:

(3) $$J^{cir}=\Lambda^{-1}J\Lambda =\left[\begin{array}{cc} A & B\\ C & D \end{array}\right]$$

where the linear‐circular transform matrix **Λ** is given as $\Lambda =\frac{1}{\sqrt{2}}\left[\begin{array}{cc} 1 & 1\\ j & -j \end{array}\right]$. For simplicity, we use *A*‐*D* to represent the four elements in the matrix, and their specific forms are shown in Section S1 (Supporting Information). For the case of circularly polarized incidence, rotating the C*m* meta‐atom from 0° to *θ* can introduce a phase shift of ΔΦ_*Geo*_ in the output light waves due to the rotational symmetry and the lattice coupling effect in the metasurfaces. The phase shift ΔΦ_*Geo*_ depends on whether *m* is odd or even. Specifically, when *m* is odd, ΔΦ_*Geo*_ = ±2*m*θ, whereas when *n* is even, ΔΦ_*Geo*_ = ±*m*θ.^[^ ^30^ ^]^ To facilitate a unified representation of the cases where *m* is odd or even, we define $n=\frac{\Delta \Phi_{Geo}}{-2\sigma \theta }$ to consistently express the ratio of phase shift to rotation angle, where *σ* is the spin state of the incident wave. Particularly, *A* and *D* in the reflection Jones matrix ***J*** *^cir^* correspond to the two polarization channels imposed with phase shifts ±ΔΦ_*Geo*_. Due to the property of high‐rotational symmetry, C*m* meta‐structures require less rotation than the common C1 or C2 ones to achieve the same geometric phase shift. Thus, once a pair of orthogonal polarization states ${|\vec{\lambda }\rangle }^{\pm }$ and required phase profiles ϕ^±^ are determined, the rotation angle *θ* of the meta‐atom can be calculated as

(4) $$\theta =-\frac{1}{4n}\left[Arg\left(A\right)-Arg\left(D\right)\right]$$

To facilitate further determination of the required propagation phase satisfying the Jones matrix in Equation (2), and avoiding complicating the Jones matrix by rotating, we can calculate the Jones matrix for the simplest case with *θ* = 0° (denoted as $J_{0}^{cir}$). When *θ* = 0°, we define the propagation phase of the meta‐atom under *x*‐polarized or *y*‐polarized incidence as *δ_x_* or *δ_y_*, respectively. For C*m* meta‐structures, independently modulating *δ_x_* or *δ_y_* by adjusting structural parameters without inducing crosstalk is challenging due to their multiple axes of symmetry. The increased symmetry complicates anisotropy control in the 2D metasurface plane. Therefore, instead of conventional *δ_y_* and *δ_x_*, we use △*δ = δ_y_–δ_x_* and Σ*δ = δ_y_* + *δ_x_* as the two key variables to characterize the meta‐atom's propagation phases.^[^ ^39^ ^]^ Finally, the Jones matrix at *θ* = 0° can be expressed in terms of △*δ* and Σ*δ* as follows:

(5) $$J_{0}^{cir}=\left[\begin{array}{cc} \cos\left(\frac{\Delta \delta }{2}\right)e^{j\frac{\Sigma \delta }{2}} & \sin\left(\frac{\Delta \delta }{2}\right)e^{j\frac{\Sigma \delta -\pi }{2}}\\ \sin\left(\frac{\Delta \delta }{2}\right)e^{j\frac{\Sigma \delta -\pi }{2}} & \cos\left(\frac{\Delta \delta }{2}\right)e^{j\frac{\Sigma \delta }{2}} \end{array}\right]$$

As shown in Equation (5), △*δ* characterizes the waveplate type associated with the meta‐structure, determining the amplitude of circularly co‐polarized and cross‐polarized components. For instance, quarter‐wave plates and half‐wave plates correspond to △*δ* = ± 90° and △*δ* = ± 180°, respectively. Σ*δ* mainly affects the phase for circularly co‐ and cross‐polarized components, as it appears in the phase factors of the matrix elements in Equation (5). Based on the above analysis, it can be straightforwardly derived that for any given pair of orthogonal polarization states ${|\vec{\lambda }\rangle }^{\pm }$ and their phase targets ϕ^±^, the rotation angle *θ* can be calculated according to Equations (1)–(4). Furthermore, the corresponding Jones matrix at *θ* = 0° can be derived based on the calculated *θ*. By simplifying this matrix to the form of Equation (5), the values of the variables △*δ* and Σ*δ* can be characterized and obtained. *θ*, △*δ* and anisotropy‐related Σ*δ* are the three key parameters that need to be physically implemented by meta‐structures. However, due to the natural limitations of high‐symmetry for C*m* meta‐structures, tailoring their anisotropy (flexibly achieving △*δ* and Σ*δ*) is more difficult than that by conventional C1 and C2 ones, which is a key challenge that must be addressed to achieve independent phase control of arbitrary orthogonal polarization states.

### 2.2 Proposed Strategy and Meta‐Atoms Performance

**Figure 2:**
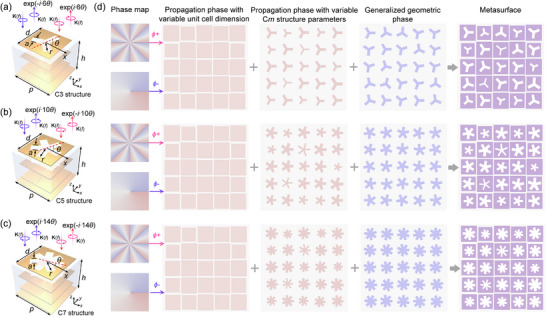
The proposed design of a) C3, b) C5, and c) C7 meta‐atoms. The period of these three meta‐atoms is *p* = 11 mm. d) Design strategy of independent wavefront tailoring using C*m* meta‐structures, where the first, second, and third columns illustrate schematic examples based on C3, C5, and C7 meta‐structures, respectively.

To overcome the difficulty in anisotropy tailoring for high‐symmetric meta‐structures, a stacked metallic meta‐atom configuration is designed, as shown in **Figure** **2**a–c. The meta‐atom is composed of four metallic layers (copper with a thickness of 0.018 mm), including three identical layers of metallic patterns and a ground layer to ensure refection operation. The three identical metallic patterns are separated by an F4B substrate with a dielectric constant of 3.5, while the third metallic pattern and the ground layer are separated by a substrate with a dielectric constant of 2.2. High‐symmetry subwavelength apertures (e.g., C3‐, C5‐, C7‐symmetry, as shown) are located on a square patch, whose size is slightly smaller than the 2D lattice period. Notably, the high‐order geometric phase shift is governed by the orientation *θ* of the C*m* aperture. To enhance anisotropy control, we perform shape tailoring to the metallic pattern while maintaining the high‐order symmetry of the subwavelength aperture. Detailed electric field simulations illustrating how the anisotropy of the meta‐atom is influenced by variations in the metallic pattern geometry are provided in Section S2 (Supporting Information). Parameters *a*, *r*, and *d* serve as the key variables to characterize the shape. As presented in the second and third columns of Figure 2d, instead of conventional propagation phase manipulation through structure parameter variation, we introduce a concept of generalized propagation phase, which includes propagation phase control through both unit cell dimension and C*m* structure parameter variation. Assisted by the variable unit cell dimension and C*m* meta‐structure parameters, flexible freedom of △*δ* and Σ*δ* manipulation can be provided. Further, once the freedoms of shape tailoring and rotation variation are combined and fully developed, the theoretical Jones matrix in Equation (2) can be physically implemented via C*m* meta‐structures. Independent phase control can be applied to any pair of orthogonal polarization states, represented in a general form of elliptically polarized waves. As the example of the orbital angular momentum meta‐generator shown in Figure 1e, topological charges of +8 and −1 are respectively generated in two orthogonal elliptically polarized channels, allowing flexible manipulation of both wavefront and polarization states. The overall on‐demand design process is summarized in Figure 2d: given wavefront targets for arbitrary orthogonal polarization states, the required propagation phase and orientation of each meta‐atom are derived from Equation (4)–(5). Shape tailoring of the unit cell and C*m* meta‐structure then provides the necessary flexibility to achieve the propagation phase target. Finally, the high‐symmetry patterns are rotated to introduce a generalized geometric phase. Through these steps, the metasurface could achieve the independent phase control for arbitrary orthogonal polarization states.

**Figure 3:**
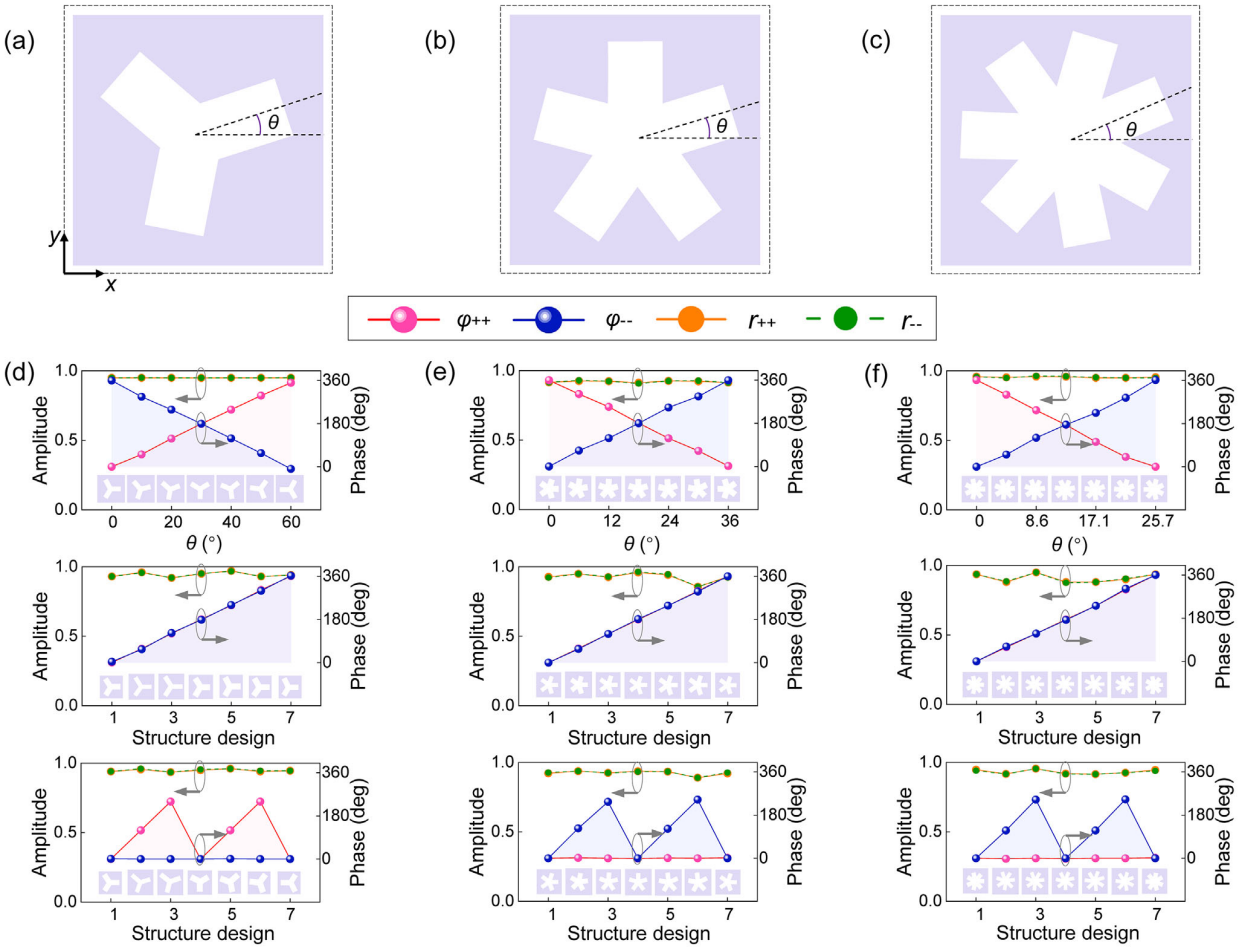
Schematic diagram and performance of meta‐structures with different high symmetry. a) C3 meta‐atom, b) C5 meta‐atom and c) C7 meta‐atom. The period of these three meta‐atoms is *p* = 11 mm. d–f): Design and performance of C3​, C5​, and C7​ meta‐atoms. The first row shows the amplitude and phase responses in the circular co‐polarization channel versus *θ*: C3 meta‐atom at 12.8 GHz with dimensions {*a*, *r*, *d*, *h*} = {2 mm, 3.75 mm, 10.8 mm, 3.5 mm}; C5 meta‐atom at 15.9 GHz, {*a*, *r*, *d*, *h*} = {1.7 mm, 3.6 mm, 10.62 mm, 8 mm}; f) C7 meta‐atom at 20.59 GHz, {*a*, *r*, *d*, *h*} = {1.94 mm, 4.1 mm, 10.58, mm, 8 mm}. The second row shows the amplitude and phase responses in the circular cross‐polarization channel versus shape. The third row illustrates the amplitude and phase responses of decoupled meta‐atoms. Parameter details of the meta‐atoms shown in the second row are provided in Section S5 (Supporting Information).

In order to validate the above design strategy, we perform full‐wave simulations for the meta‐atoms using CST Microwave Studio. Theoretically, the rotation of C3, C5, and C7 apertures (**Figure** **3**a–c) with an angle of *θ* can respectively provide geometric phase shifts of ± 6*θ*, ∓ 10*θ* and ∓14*θ* for a pair of circularly polarized waves.^[^ ^30^ ^]^ The top row of Figure 3d–f shows the simulated reflection amplitude responses (denoted as *r*_++_ and *r*_−_) and phase responses (denoted as *φ*_++_ and *φ*_−_), where “+” and “−” indicate left‐handed circular polarization (LCP) and right‐handed circular polarization (RCP), respectively. For the C3, C5, and C7 meta‐atoms, rotations of 60°, 36°, and 25.7°, respectively achieve a full 360° phase coverage while maintaining a high amplitude of over 0.9. Here, the theoretical predictions for the phase responses of ± 6*θ*, ∓ 10*θ* and ∓ 14*θ* are well demonstrated through simulation. Additionally, the middle row of Figure 3d–f illustrates results for achieving a propagation phase gradient, with each meta‐atom designed as a half‐wave plate, achieving full 360° phase coverage with high efficiency solely through shape tailoring. The full‐wave simulation results are in agreement with the design targets, confirming the phase manipulation capabilities of the designed C*m* meta‐atoms. Notably, the achieved propagation phase coverage resulted from both variable unit cell dimension and C*m* meta‐structure parameters, significantly enhancing the phase coverage. Additional simulation details on parameter variation are provided in Section S3 (Supporting Information). In addition, the strategy of stacking multiple metallic layers within the meta‐atom can significantly expand the phase modulation range compared to a single‐layer configuration, as detailed in Section S4 (Supporting Information). Furthermore, we combine the orientation rotation shown in the first row of Figure 3d–f and the shape tailoring shown in the second row to obtain the series of meta‐atoms illustrated in the insets of the third row. It is found that the phases of the pair of spin states are well decoupled, which is in agreement with the theoretical predictions in the abovementioned section. It should be noted that the results presented in Figure 3 correspond to a single operating frequency. In practice, the operational bandwidth of the meta‐atoms can be further extended by increasing the substrate thickness, adding additional metallic layers, or introducing asymptotically varied structural parameters in the metal pattern design, which offer promising chances for broadband phase manipulation.

### 2.3 Demonstration of Polarization‐Multiplexing Meta‐Devices

**Figure 4:**
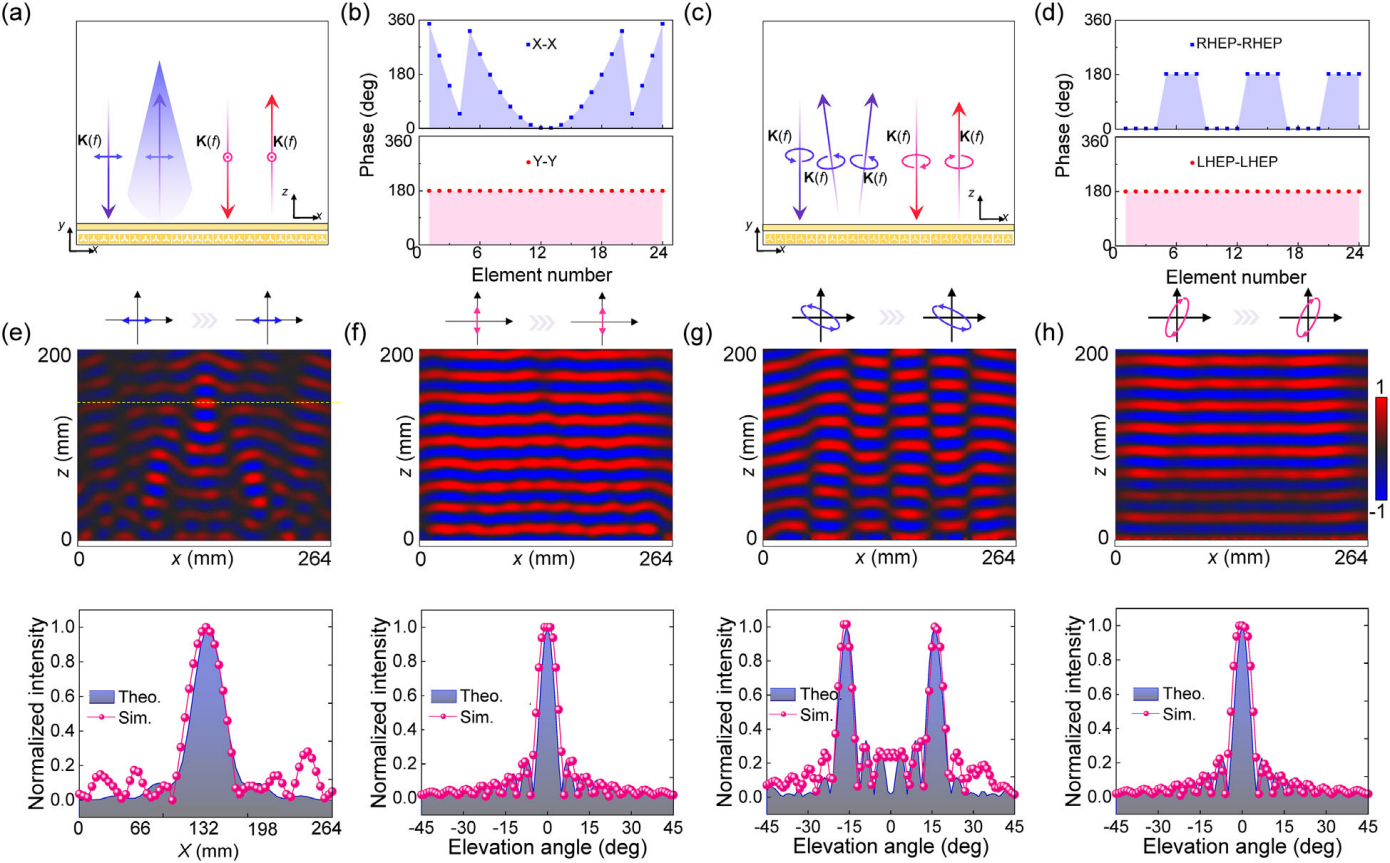
Design and performance of linear‐ and elliptical‐polarization‐decoupled meta‐devices. a) Conceptual illustration of a linear‐polarization‐selective meta‐lens, and b) decoupled phase profiles in a pair of orthogonal polarization channels (*x*‐polarized and *y*‐polarized). c) Conceptual illustration of an elliptical‐polarization‐selective meta‐splitter, and d) decoupled phase profiles for orthogonal elliptical polarization channels. e,f): Performance of the linear‐polarization‐selective meta‐lens under *x*‐ and *y*‐polarized illumination, respectively. The first row shows the simulated reflected electric field distribution; the second row of shows the 1D near‐field intensity at the focal length, while the second row shows the far‐field scattering pattern. g,h): Performance of the elliptical‐polarization‐selective meta‐splitter under orthogonal elliptical polarization illumination. The first row shows the simulated reflected electric field distribution, and the second row shows the corresponding far‐field scattering patterns.

To validate phase control for various polarized waves at the device level, we first design two polarization‐selective meta‐devices based on C3 meta‐structures and demonstrate their functions in the near and far field. These devices decouple the wavefront of a pair of orthogonal linearly polarized and elliptically polarized light waves. The orthogonal linear polarization states are determined by *α* = 0° and *χ* = 0° according to Equation (1), corresponding to the commonly termed *x*‐ and *y*‐polarized waves. **Figure** **4**a schematically shows the linear‐polarization‐selective meta‐lens, which can selectively focus the illuminated *x*‐polarized wave while reflecting the *y*‐polarized one. The decoupled phase profiles are clearly shown in Figure 4b, with the focal length set to 150 mm. For a pair of elliptical polarization states, a polarization‐selective meta‐splitter is designed and demonstrated. Herein, we randomly set *α* = 60° and *χ* = −108° to define the two orthogonal elliptical polarization states. As depicted in Figure 4c, the prototype serves as a beam splitter for right‐handed elliptically polarized incidence, deflecting the incident beam to directions of ±18° in the *x‐z* plane. Differently, under the left‐handed elliptically polarized illumination, the device exhibits a uniform phase distribution and operates as a standard reflector. The specific phase profiles in these two orthogonal elliptically polarized channels are shown in Figure 4d, distributed along the *x*‐direction. Both the linear‐polarization‐selective meta‐lens and the elliptical‐polarization‐selective meta‐splitter are composed of 24 × 24 meta‐atoms, for which more details of these two meta‐devices are provided in Section S6 (Supporting Information). To further validate the phase‐decoupling performance, we perform full‐wave simulations of these two meta‐devices and observe the co‐polarized reflected field. The simulated near‐field results in Figure 4e–h meet the design goals for linear‐polarization‐selective focusing and elliptical‐polarization‐selective splitting, and more details are further present in the second row. In the second row of Figure 4e, we compare the simulated profile of the focusing spot at designed *z* = 150 mm with the theoretical profile. Simulated far‐field scattering patterns corresponding to Figure 4f–h are also compared with theoretical results in the second row of them. The simulated results are in well agreement with the theoretical predictions, effectively demonstrating the decoupling of wavefront control for a pair of orthogonal linear or elliptical polarization states. In addition to the above‐validated polarization‐selective meta‐devices, two polarization‐multiplexed metasurface designs including a linear‐polarization‐multiplexed meta‐reflector and an elliptical‐polarization‐multiplexed meta‐lens are further proposed and demonstrated. These devices respectively steer a pair of orthogonal linearly polarized waves into different directions and focus a pair of orthogonal elliptically polarized waves to distinct spatial positions, thereby validating the proposed method's capability for independent and flexible wavefront tailoring across various orthogonal polarization states, as detailed in Section S7 (Supporting Information).

**Figure 5:**
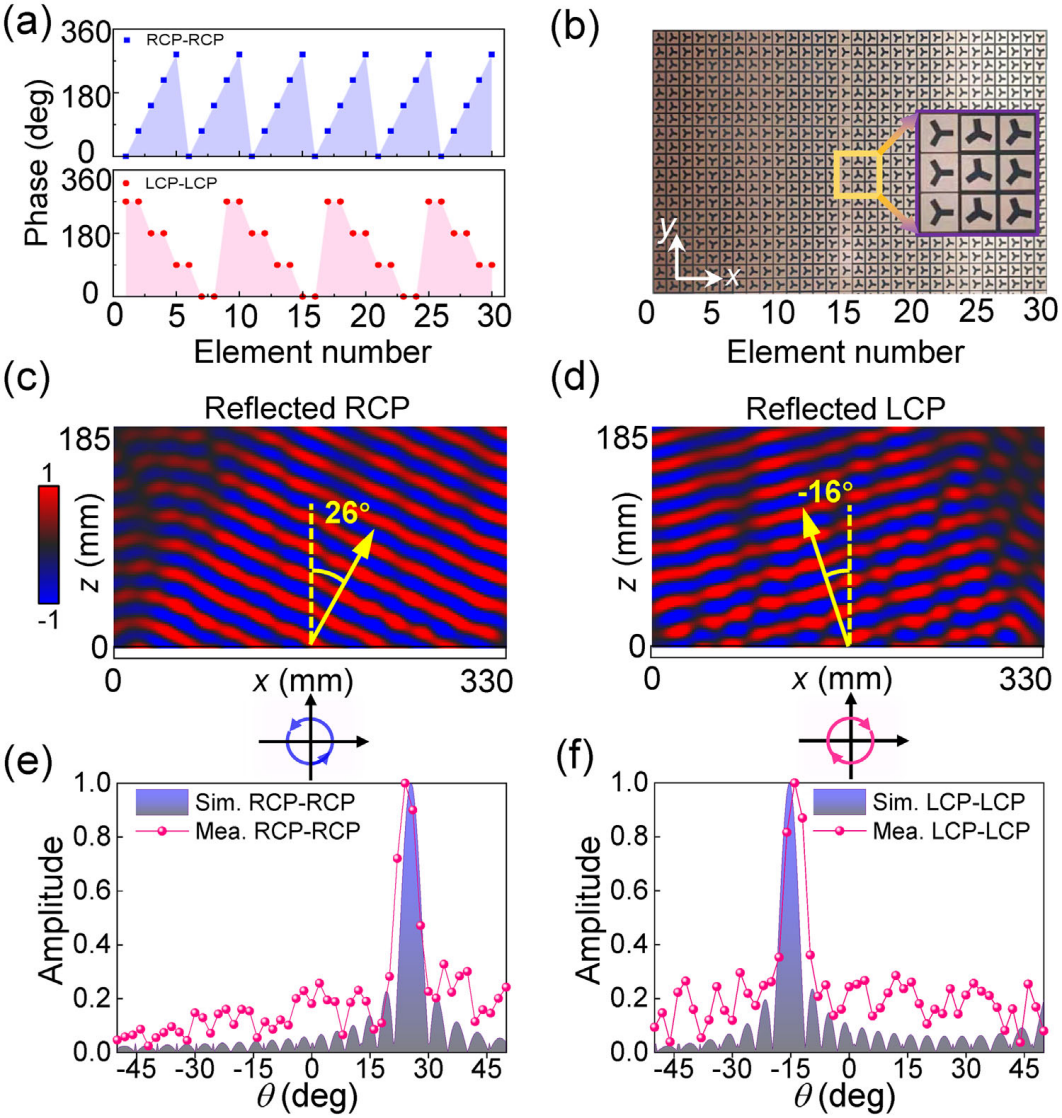
Design and experimental demonstration of the spin‐decoupled beam meta‐deflector. a) 1D decoupled phase profiles for the RCP‐RCP and LCP‐LCP channels along the *x*‐direction (the polarization components before and after the “−” represent the incident and reflected polarizations, respectively). b) Photograph of the fabricated prototype. Simulated reflected electric field distributions under c) RCP and d) LCP wave illumination. e,f) Measured and simulated normalized scattering intensity versus the deflection angle for RCP and LCP incidences.

Furthermore, we demonstrate that the wavefronts of orthogonally polarized waves can be tailored for specific functionalities and verify them experimentally. As an example, we use a pair of orthogonal circularly polarized waves for illustration, which are commonly employed in practical applications. For this case, with *α* = 90° and *χ* = 45°, we realize and experimentally demonstrate a spin‐multiplexing meta‐deflector based on C3 meta‐structures. From Equation (5), for a pair of circularly polarized waves, the co‐polarized component should be controlled to zero to ensure highly efficient cross‐polarization conversion, resulting in △*δ* = 180° in Equation (5).^[^ ^38^ ^]^ Additionally, the phase responses can be briefly described by *ϕ* ^±^ = 0.5∑*δ* ± 2*θ*. When *ϕ* ^+^ and *ϕ* ^−^ are independently modulated, the meta‐atoms in the device exhibit a variety of ∑*δ* responses while consistently maintaining △*δ* = 180°, equivalent to half‐wave plates with various initial phases. Specifically, the meta‐deflector is composed of 30 × 30 half‐wave‐plate elements, with 1D phase gradients of *dφ*/*dx* = 72° and *dφ*/*dx* = −90° in the two orthogonal circular polarization channels, respectively. The discretized phase profiles for both circular polarization channels are presented in **Figure** **5**a. This spin‐multiplexing meta‐deflector, fabricated using printed circuit board technology, has an overall dimension of 330 mm × 330 mm (Figure 5b). According to the phase gradient, a deflection angle of 26° for normal RCP incidence and −16° for normal LCP incidence in the *x‐z* plane can be calculated by the generalized Snell's law.^[^ ^40^ ^]^ As depicted in Figure 5c,d, the simulated electric field distributions confirm that two independent phase gradients are imparted to the reflected waves. Measurement of the fabricated prototype is conducted in a microwave anechoic chamber (the schematic diagram of the experiment setup is provided in Section S8, Supporting Information). As clearly depicted in Figure 5e,f, the experimental far‐field results align well with the simulated ones. The anomalous deflection angles for RCP and LCP are effectively decoupled, rather than being limited to two opposite deflection angles, enabling flexible and independent wavefront control of reflected waves with different spin states via C*m* meta‐structures. In addition to the C3 meta‐structures, two C5‐based meta‐devices are also designed and analyzed using full‐wave simulations. The first device achieves selective beam splitting for a pair of linear polarizations, while the second focuses energy at different focal lengths under illumination by different circularly polarized waves (Section S9, Supporting Information), demonstrating the versatile wavefront tailoring capacities of high‐symmetry meta‐structures.

**Figure 6:**
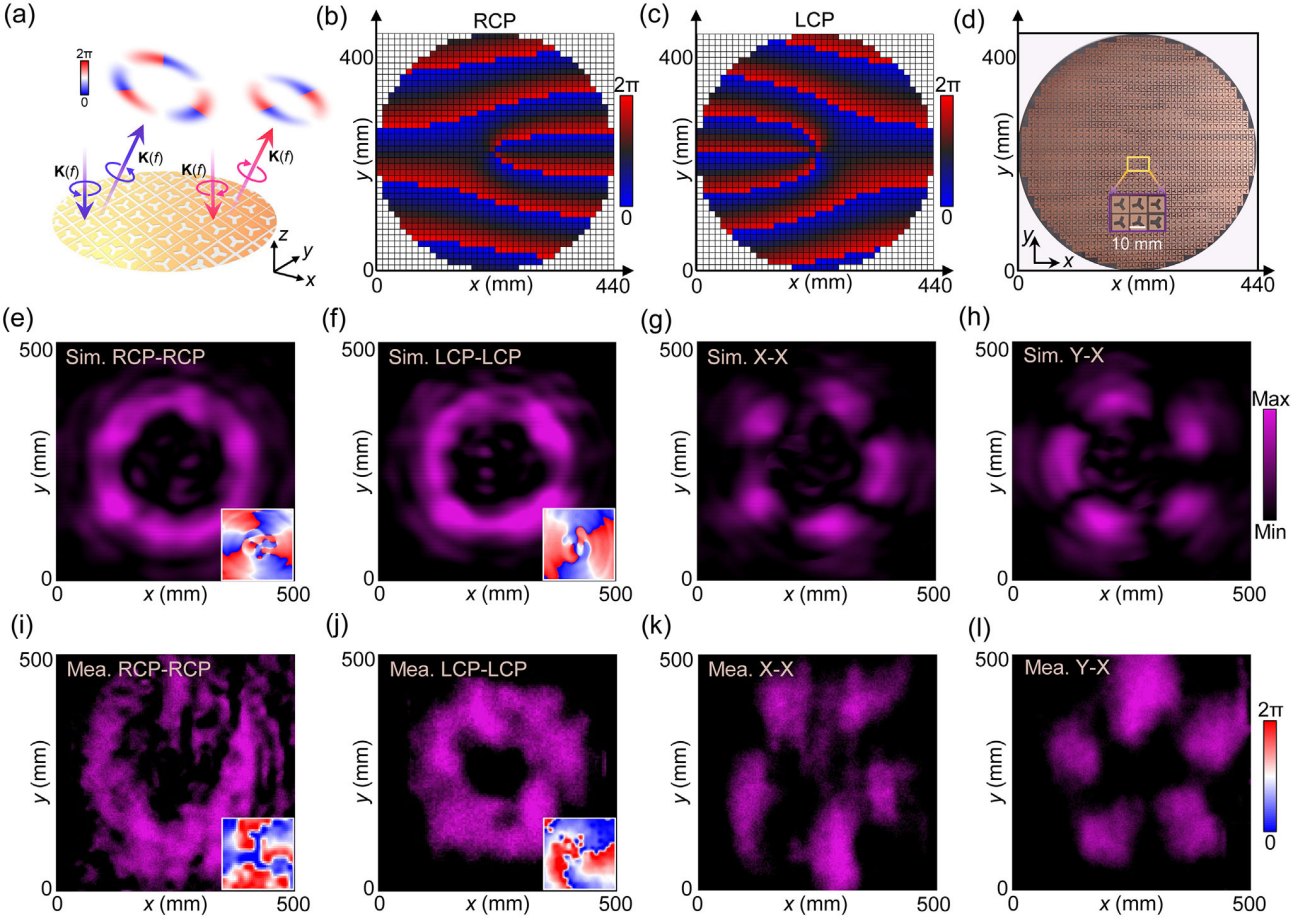
Design and results of the arbitrary spin‐to‐orbital angular momentum convertor based on meta‐structures with C3 rotational symmetry. a) Conceptual illustration of the meta‐convertor. Phase profiles for off‐axis vortex beams with topological charges of b) +3 in the RCP‐RCP channel and c) −2 in the LCP‐LCP channel. d) Photograph of the fabricated prototype, illuminated by a planar *x*‐polarized light wave at 12.8 GHz. Simulated electric intensity and phase distributions of e) RCP, f) LCP, g) *x*‐polarized, and h) *y*‐polarized components in the reflective field. i–l) Measured electric intensity and phase distributions of the corresponding four components. The polarization states on the left and right sides of the “−” symbol represent the observed polarization state and the incident polarization state, respectively.

Converting spin angular momentum to vortex beams carrying orbital angular momentum has significant applications in optics and photonics.^[^ ^41^, ^42^, ^43^ ^]^ Conventional optical elements for this conversion suffer from spin coupling and can only generate orbital angular momentum with opposite states for a pair of orthogonal spin waves.^[^ ^44^, ^45^ ^]^ In 2017, R. C. Devin et al. proposed an approach for arbitrary spin‐to‐orbital angular momentum conversion by combining *δ_x_*, *δ_y_*, and *θ*.^[^ ^46^ ^]^ However, this approach has not been proven extendable to C*m* meta‐structures. Here, we use a C3 meta‐structure as an example and experimentally demonstrate its capacity to manipulate complex light fields. As schematically shown in **Figure** **6**a, off‐axis vortex beams with topological charges of +3 and −2 are generated when the metasurface is illuminated by planar waves carrying spin angular momentum *σ* = +1 and −1, respectively. Figure 6b,c show the calculated phase distributions required for these two spin channels. To avoid the blockage of the reflected beams by the transmitting antenna, these two vortex beams are designed to have the same deflection angles of 16° in the *y‐z*‐plane. As shown in Figure 6d, the fabricated meta‐convertor comprises ≈1200 meta‐atoms. Figure 6e,f presents the simulated electric field distribution of RCP and LCP components, which exhibit apparent donut‐shape intensity and spiral phase distribution with orbital angular momentum having topological charges of +3 and −2. Notably, when vortex beams carrying different orbital angular momentum modes propagate in the same direction, the inhomogeneous polarization state can be characterized by their coherent superposition ^[^ ^47^ ^]^ and described using the high‐order Poincaré sphere.^[^ ^44^, ^48^ ^]^ When a pair of orthogonal linearly polarized components in the reflected field are detected separately, several nulls appear in the donut‐shaped energy distributions for each single linear polarization component. The number of these nulls can be calculated as |*m*‐*n*| = 5,^[^ ^49^ ^]^ which is well validated in the simulated results in Figure 6g,h. For further experimental demonstration, a wideband linearly polarized antenna is used to transmit an *x*‐polarized wave onto the meta‐convertor, and the reflected electric field is observed in the off‐axis output plane using a near‐field scanning system (details in Figure S6b in Section S5, Supporting Information). The measured results, presented in Figure 6i–l, clearly characterize the orbital angular momentum modes of +3 and −2, closely matching both the design targets and the simulation results. The slight deviations observed in the experimental results are mainly caused by fabrication tolerances and residual interference from the incident field during reflective field measurements. In brief, a decoupled spin‐to‐orbit angular momentum convertor, particularly based on a C*m* meta‐structure, is experimentally demonstrated, highlighting the powerful potential of C*m* meta‐structures for advanced wavefront tailoring.

## 3 Conclusion

In summary, we present a generalized method to independently tailor the wavefront of arbitrary orthogonal polarized waves via C*m* (*m* ≥ 3) meta‐structures, establishing a potential foundation for wavefront engineering in high‐symmetry meta‐structures. This method can significantly enhance the anisotropy tailoring and phase control capabilities of high‐symmetry meta‐structures, offering substantial potential for integrating wavefront engineering with exotic optical responses. By considering shape tailoring of the meta‐atom, including both unit cell dimension and C*m* meta‐structure parameters, we achieve substantial control over the generalized propagation phase. The further combination with the high‐order geometric phase provides exciting opportunities for wavefront engineering across various polarization states. For validation and application, a series of polarization‐multiplexing meta‐devices are numerically and experimentally demonstrated for reliability and robustness. In contrast to conventional approaches only applicable for C1 and C2 meta‐structures, this method breaks the inherent impression that high‐symmetry meta‐structures lack potential in independent wavefront engineering of dual‐spin waves. Moreover, to achieve the same phase modulation, high‐order symmetric meta‐atoms require smaller rotation angles than C1 and C2 meta‐structures. This is beneficial for metasurfaces with non‐uniform phase profiles, as smaller orientation differences between adjacent meta‐atoms may improve the structural uniformity and suppress the near‐field coupling, potentially enhancing the overall robustness of the device performance. This work is expected to inspire further innovations and applications in metamaterial technologies utilizing structures with high symmetry,^[^ ^50^ ^]^ and may be potentially extendable to other fields such as acoustics, thermotics, and mechanics.

## Conflict of Interest

The authors declare no conflict of interest.

## Supporting information



Supporting Information

## Data Availability

The data that support the findings of this study are available from the corresponding author upon reasonable request.
