# Supplementary material for: Generalized Phase Tailoring of Arbitrary Orthogonal Polarizations in Meta‐Structure with High‐Order Geometric Symmetry
Source: Adv Sci (Weinh). 2025 May 14;12(28):2504918. doi: 10.1002/advs.202504918 (PMC12302587; doi:10.1002/advs.202504918)
Supplement: Supplementary file 1 — Supporting Information [file ADVS-12-2504918-s001.docx]

Supporting Information

Generalized Phase Tailoring of Arbitrary Orthogonal Polarizations in Meta-Structure with High-Order Geometric Symmetry

*Kai Qu, Ke Chen,* Qi Hu, Weixu Yang, Junming Zhao, Tian Jiang and Yijun Feng**

K. Qu, K. Chen, Q. Hu, W. Yang, J. Zhao, T. Jiang, Y. Feng

School of Electronic Science and Engineering,

Nanjing University

Nanjing 210023, China

E-mail: ke.chen@nju.edu.cn; yjfeng@nju.edu.cn

J. Zhao, Y. Feng

Suzhou Laboratory

Suzhou 215000, China

Contents

| S1. Derivation about the Jones matrix for independent phase control of arbitrary orthogonal polarization states ………………………………………………………………………………………………………… | 2 |
| --- | --- |
| S2. Simulated electric field distribution of meta-atom with linearly polarized incidence……………... | 2 |
| S3. Propagation phases from the C*m* meta-structure and the unit cell ………………………......................... | 3 |
| S4. Comparison between different layers of the meta-atom’s configuration………………………………. | 7 |
| S5. Parameter details about the meta-atom examples with different symmetries ………………………... | 8 |
| S6. Details of linear/elliptical-polarization-selective meta-devices ……………………………………........... | 8 |
| S7. Design of linear/elliptical-polarization-multiplexing meta-devices……………………………............... | 9 |
| S8. Experiment setup of the meta-device measurement…………………………………………………………… | 12 |
| S9. C5-based polarization-multiplexing meta-devices……………………………………………………………… | 12 |

**S1. Derivation about the Jones matrix for independent phase control of arbitrary orthogonal polarization states**

To intuitively express the independent phase control for arbitrary orthogonal polarization states, we use |${\vec{\text{λ}}\rangle}^{\text{±}}$ |${\vec{\text{λ}}\rangle}^{\text{±}}$ to describe the process, where |${\vec{\text{λ}}\rangle}^{\text{±}}$ are the two polarization states and are the independent phase profiles need to be imposed on them. Herein, we characterize the orthogonal polarization states in the form of the Jones vector on the basis of linear polarization: |${\vec{\text{λ}}\rangle}^{\text{+}}=\left[ \begin{matrix} \text{cos}\text{ }\text{χ} \\ \text{e}^{\text{i}\text{α}}\text{sin}\text{ }\text{χ} \end{matrix} \right]$, |${\vec{\text{λ}}\rangle}^{\text{-}}=\left[ \begin{matrix} \text{cos}\text{ }\text{χ} \\ \text{e}^{\text{i}\text{α}}\text{cos }\text{χ} \end{matrix} \right]$. Where *α* and χ set the polarization states. The output electric field can be calculated by the matrix as [|${\vec{\text{λ}}\rangle}^{\text{+}}\text{e}^{\text{i}\text{ϕ}^{\text{+}}} |{\vec{\text{λ}}\rangle}^{\text{-}}\text{e}^{\text{i}\text{ϕ}^{\text{-}}}]=\text{J }\left[ |{\vec{\text{λ}}\rangle}^{\text{+}} |{\vec{\text{λ}}\rangle}^{\text{-}} \right]$. This process can be further written as ^[1]^:

(S1)

Where ***J*** is the Jones matrix on the basis of linear polarization, and it can be calculated as:

(S2)

According to the linear-circular transform matrix **Λ**$\text{=}\frac{\text{1}}{\sqrt{\text{2}}}\left[ \begin{matrix} \text{1} & \text{1} \\ \text{j} & \text{-j} \end{matrix} \right]$, the Jones matrix can be further written in the form of circular polarization basis:

$J^{cir}=\Lambda^{-1}J\Lambda=\left[\begin{matrix} A & B \\ C & D \end{matrix}\right]$ (S3)

**S2. Simulated electric field distribution of meta-atom with linearly polarized incidence**

In this section, full-wave simulations of the electric field distributions are conducted to show the operating modes of meta-atoms under linearly polarized wave incidence. Figures S1(a) and S1(b) present the simulated reflection spectra of an isotropic meta-atom and an anisotropic meta-atom, respectively. Figures S1(c) and S1(d) show the *y*-polarized and *x*-polarized electric field distributions of the isotropic C3 meta-atom, with the rotation angle set to 0°. It is evident that under *y*-polarized incidence, the electric field is concentrated in the lobe of the C3-shaped metallic aperture along the *x*-direction, while under *x*-polarized incidence, the field is primarily distributed in the two lobes aligned with the *y*-direction. Notably, the field intensities under the two orthogonal linear polarizations are comparable, consistent with the intrinsic isotropy of the meta-atom. For the anisotropic meta-atom, the spatial patterns of the electric field distributions under *y*- and *x*-polarized incidence remain similar to those of the isotropic case; however, a marked difference in field intensity is observed, as shown in Figs. S1(e) and S1(f). This discrepancy arises from the stronger resonance excited by the *y*-polarized wave, which induces enhanced surface currents. As a result, the meta-atom exhibits pronounced anisotropy in its electromagnetic response.


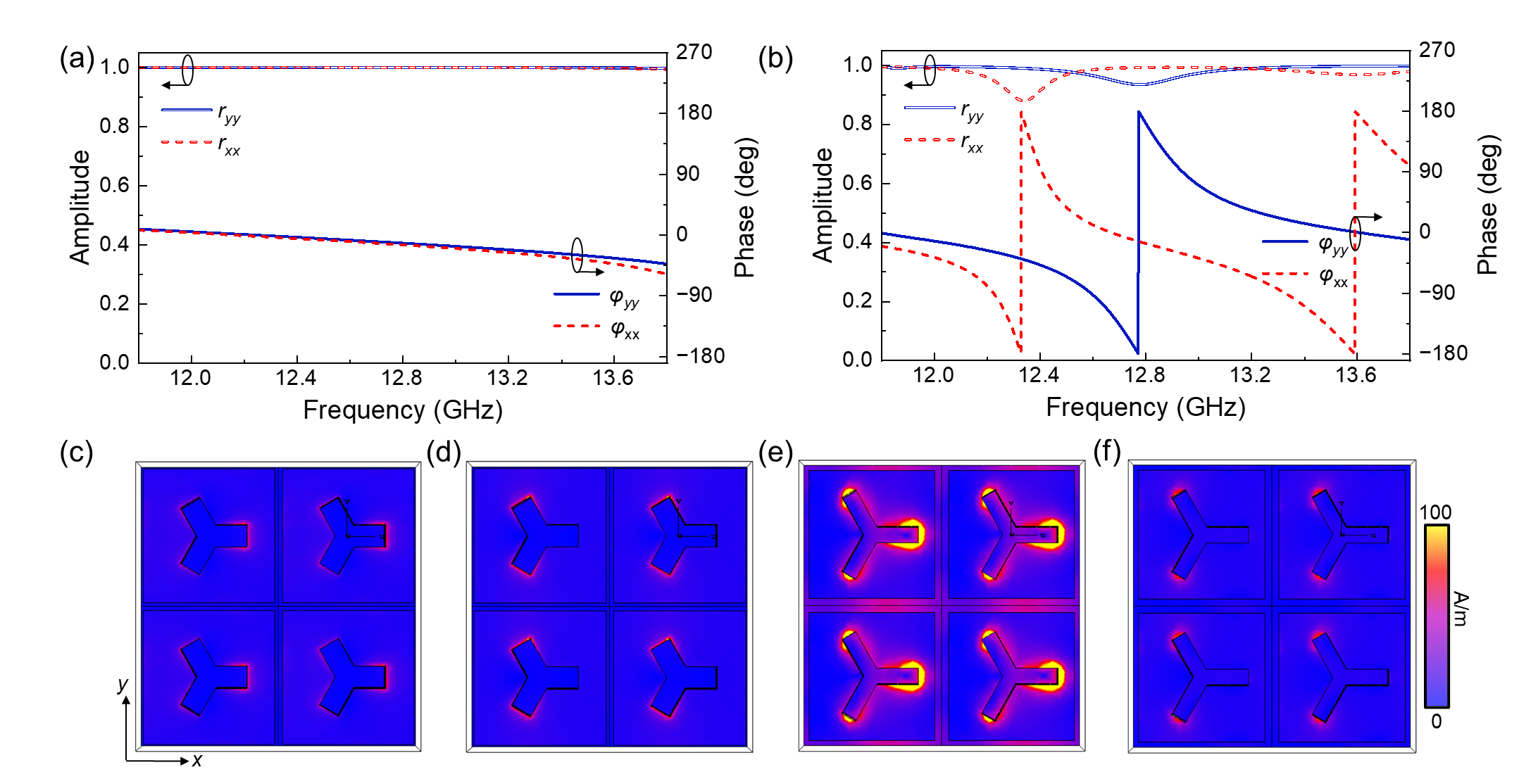


**Figure S1**. Simulated reflection spectra and electric field distributions for isotropic and anisotropic C3 meta-atoms. (a) Reflection spectrum of the isotropic C3 meta-atom with structural parameters {*a*, *d*, *r*} = {1.75 mm, 10.4 mm, 3 mm}. (b) Reflection spectrum of the anisotropic C3 meta-atom with structural parameters {*a*, *d*, *r*} = {1.25 mm, 9.9 mm, 3.67 mm}. Electric field distribution of the isotropic C3 meta-atom respectively under (c) *y*-polarized and (d) *x*-polarized illumination at 12.8 GHz. Electric field distribution of the anisotropic C3 meta-atom respectively under (e) *y*-polarized and (f) *x*-polarized illumination at 12.8 GHz.

**S3. Propagation phases from the C*m* meta-structure and the unit cell**

For sub-wavelength structures with a fixed rotation angle, adjusting their shape by changing physical parameters results in phase shifts in their linearly polarized channels. This kind of phase shift is known as propagation phase or dynamical phase. Most current works with propagation phase shifts are based on C2​ structures, which have two symmetry axes in orthogonal directions, providing natural advantages in modulating the propagation phases in two orthogonal linear polarizations. Specifically, this orthogonality enables the avoidance of crosstalk between the two linear polarizations when modulating their propagation phases. For example, in a cross resonator, the propagation phase is determined solely by the length of the arm in the corresponding direction. Consequently, propagation phase in previous works is mainly achieved by the shape adjustment of the structure that needs to be rotated (as shown in Fig. S2).


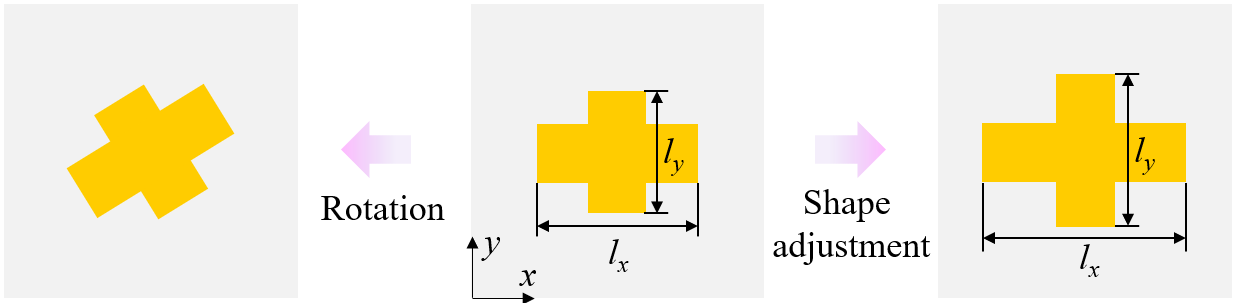


**Figure S2.** Schematic diagram of the approach for offering propagation phase shift via C2 structure. In the cross resonator, *l_x_* and *l_y_* are the lengths of arms in the *x-* and *y-* directions, respectively. The orange and grey structures represent the resonator and dielectric substrate, respectively.

But for C*m* (*m* ≥ 3) meta-structures, in addition to obtaining a relatively wide propagation phase coverage, there are two conditions that also need to be met when adjusting the shape of the structure: (1) the high symmetry should be maintained, for which the high-order geometric phase shift can be fixed as the stable linear function of rotation angle; (2) the anisotropy of the structure should be well maintained with the variation of the shape parameters. In other words, when rotation angle is fixed as 0°, there should be an almost constant phase difference (denoted as △*δ*) between the linearly polarized propagation phase in *x* direction (denoted as *δ_x_*) and the propagation phase in *y* direction (denoted as *δ_y_*). For instance, with regard to the structure of half-wave plate, △*δ* should be fixed around 180° at the operating frequency.

Herein, we take C3 sub-wavelength aperture as an example ^[2]^. For a meta-atom with an equilateral triangle-shaped aperture, there is only one degree of freedom (the distance from the vertex to the rotation center, denoted as *r*) to modulate the propagation phase, which is not conductive to achieving a large phase coverage with variations in the aperture shape. In contrast, the Y-shaped aperture (the meta-atom discussed in the main text) has more degrees of freedom for shape adjustment, as illustrated in Fig. S3.


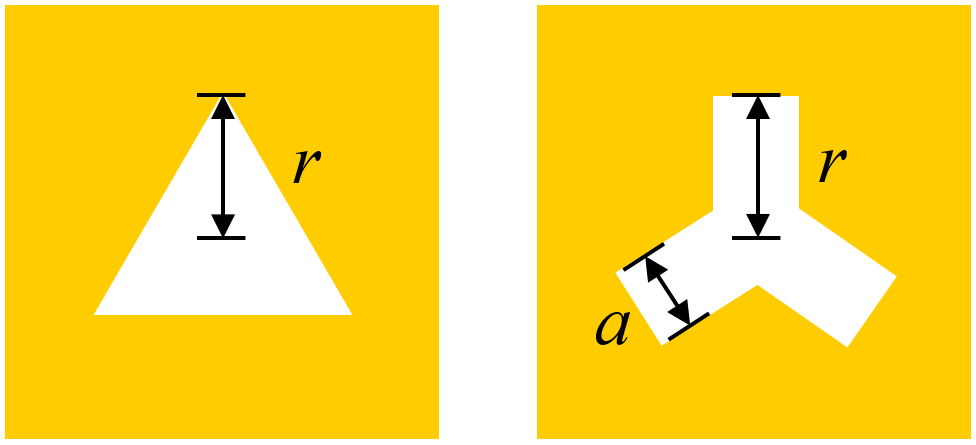


**Figure S3**. Schematic diagrams of equilateral-triangle-shaped and Y-shaped sub-wavelength aperture. *a* is the width of Y-shaped aperture.

Herein, we introduce propagation phase in the C3 aperture parameters (shown in Fig. S4(a)) and in the unit cell dimension (shown in Fig. S4(b)).


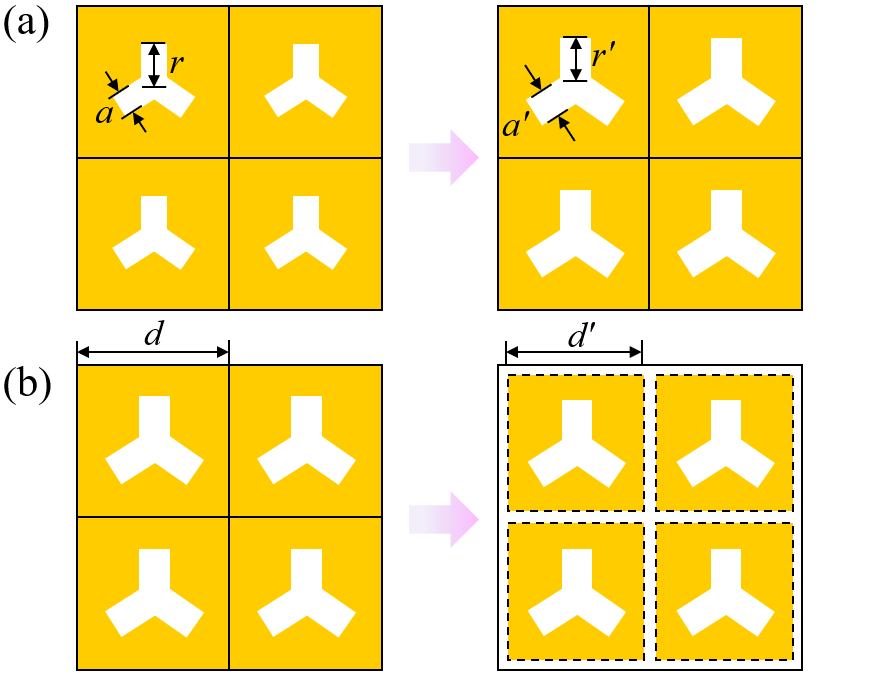


**Figure S4.** Schematic diagrams of propagation phase (a) in the C3 aperture parameters and (b) in the unit cell dimension.

When the physical parameters {*a*, *r*} are adjusted to {*a’*, *r’*}, the high symmetry of the Y-shaped aperture can be well maintained. But the variation of *a* and *r* can only offer limited propagation phase coverage. As shown in Figs. S5 (a)-(d), when *a* varies in the range of 3.7-3.9 mm or *r* varies in the range of 1.6-2 mm, the coverage of propagation phase is no more than 100° under the illumination of circularly polarized wave. Although increasing the range of variation in these physical parameters can further enhance the coverage of the propagation phase shift, its amplitude decreases rapidly.


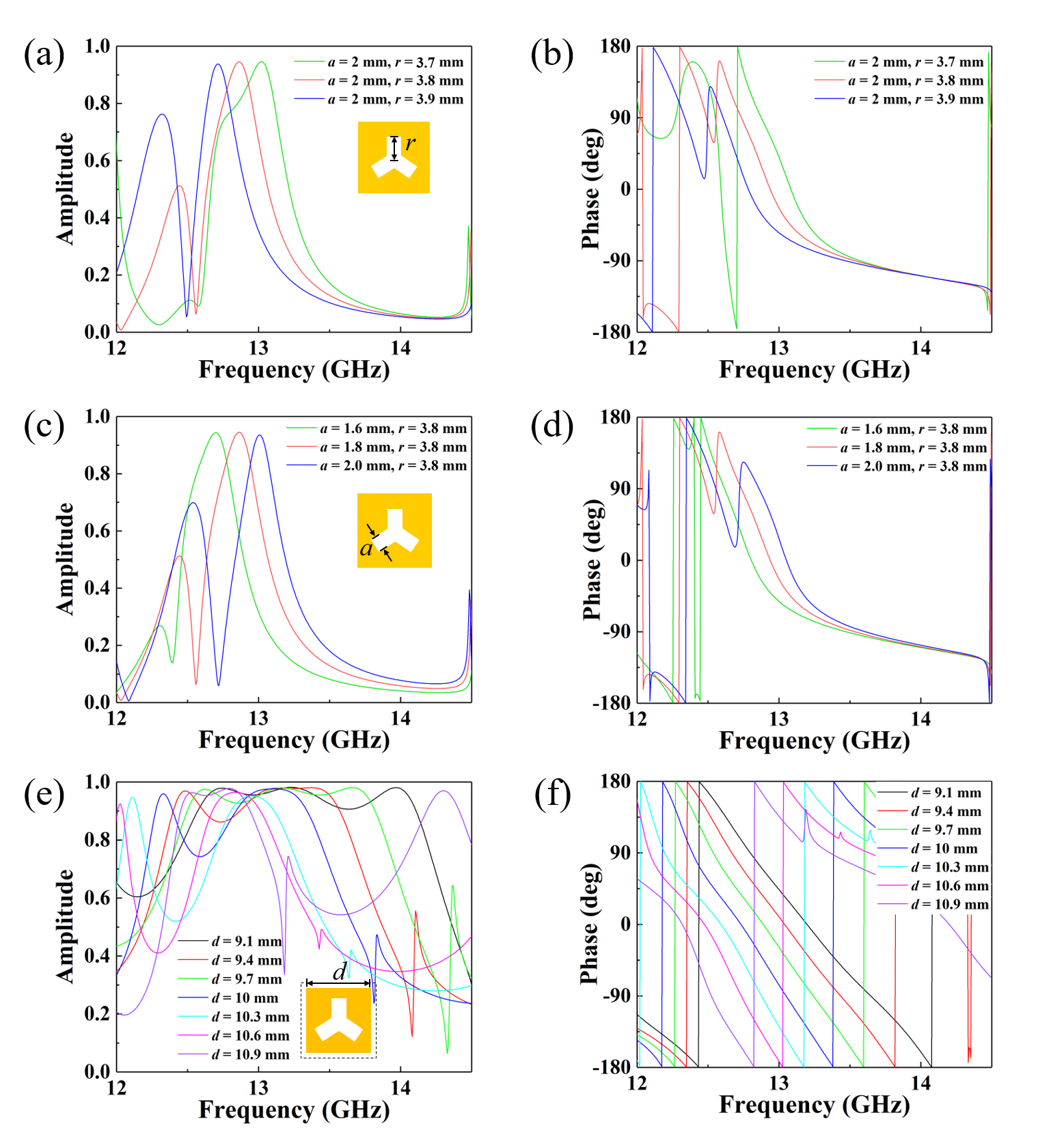


**Figure S5.** Simulated relationships between the circularly co-polarized amplitude/phase response and physical parameters {*a*, *r*, *d*}. Co-polarized (a) amplitude and (b) phase responses versus frequency and *a*. Other physical parameters of the meta-atom in the inset in (a) are: *a* = 2 mm, *d* = *p* =11 mm. Co-polarized (c) amplitude and (d) phase responses versus frequency and *a*. Other physical parameters of the meta-atom in the inset in (c) are: *r* = 3.8 mm, *d* = *p* =11 mm. Co-polarized (e) amplitude and (f) phase responses versus frequency and *d*. Other physical parameters of the meta-atom in the inset in (c) are: *r* = 3.8 mm, *a* = 2 mm.

Compared to *a* and *r*, the variation of *d* possesses a superior capability for propagation phase modulation and stability in maintaining high amplitude. As shown in Figs. S5(e)-(f), when *d* varies in the range of 9.1-10.9 mm and other physical parameters are fixed, a wide propagation phase coverage can be obtained. Additionally, under these conditions, the reflection amplitude consistently remains above 0.9.

Since *a* and *r* determine the basic size of the Y-shaped aperture, which is rotated to bring about a high-order geometric phase, we refer to this part of propagation phase shift (brought by the variation of *a* or *r*) as propagation phase with variable C*m* meta-structure parameters. Likewise, *d* determines the size of the unit cell, which is not rotated with the Y-shaped aperture. And we call this part of propagation phase shift (brought by the variation of *d*) as propagation phase with variable unit cell dimension. Although changing only *d* can also meet the requirements for propagation phase control in spin-decoupled phase control, the Y-shaped aperture will exceed the boundary of the square metallic structure when *d* is small enough. Therefore, we employ a strategy that combines these two parts to achieve a full propagation phase coverage of 360°, enabling the formation of various wave plates to realize independent phase control for arbitrary orthogonal linear and elliptical polarization states.

**S4. Comparison between different layers of the meta-atom’s configuration**

In this section, to illustrate the advantage of the multilayer configuration in the proposed meta-atom design, full-wave simulations are conducted to evaluate the propagation phase coverage under identical variations of structural parameters. As shown in Fig. S6(a), when the structure is varied from a randomly selected structure 1 to structure 2, only a slight change is observed in the co-polarized reflection phase spectrum under *x*-polarized incidence for the single-layer meta-atom. However, when the same structural parameters are applied to a dual-layer configuration, the phase shift resulting from the structural variation becomes significantly more pronounced, as depicted in Fig. S6(b). Furthermore, by increasing the configuration to a three-layer structure, the achievable propagation phase range is further extended under the same parameter change, as shown in Fig. S6(c). These results clearly demonstrate that multilayer meta-atom configurations offer enhanced tunability and greater potential for achieving broad propagation phase modulation through structural parameter variation.


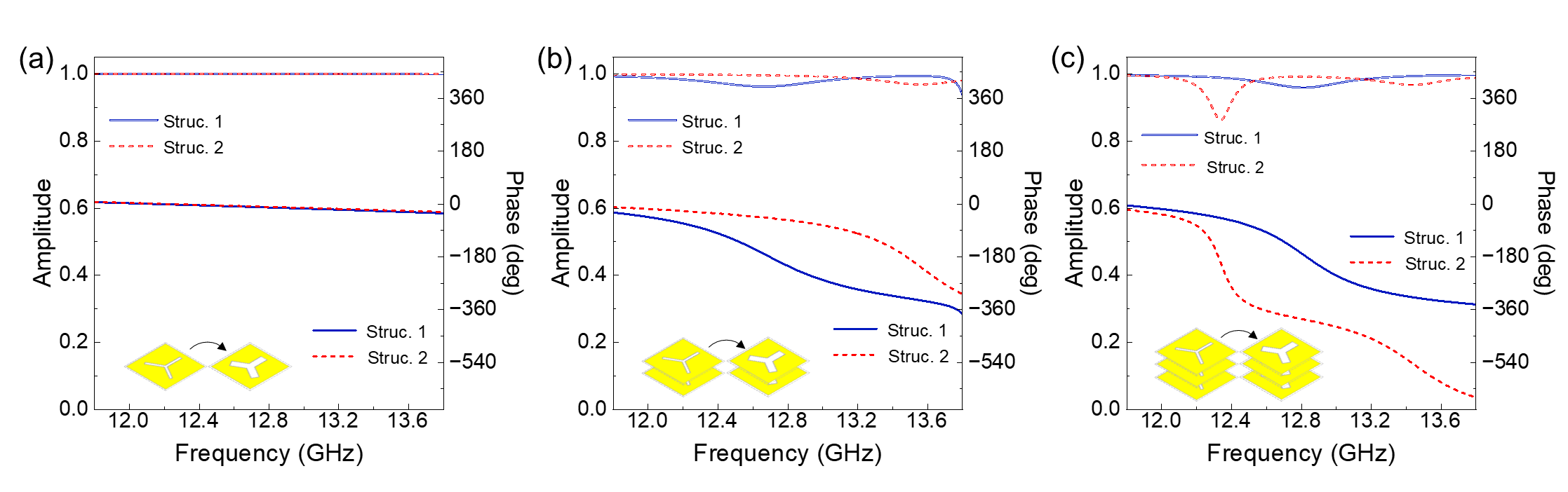


**Figure S6**. Comparison of simulated *x*-polarization phase shifts resulting from varying the same set of structural parameters under different layer configurations: (a) single-layer; (b) dual-layer; (c) tri-layer. The meta-atoms shown in the insets illustrate the variation of parameters {*a*, *d*, *r*} from {0.5 mm, 10.4 mm, 4 mm} to {1.5 mm, 10.15 mm, 3.67 mm}.

**S5. Parameter details about the meta-atom examples with different symmetries**

In this section, Tables S1-3 present parameter details of meta-atoms shown in Figs. 2(g)-(i).

Table S1. Parameter details of C3 meta-atoms shown in Fig. 2(g)

| Structure sequence | *a* (mm) | *r* (mm) | *d* (mm) | *θ* (°) |
| --- | --- | --- | --- | --- |
| 1 | 2.00 | 3.70 | 9.50 | 0 |
| 2 | 1.70 | 4.10 | 10.60 | 0 |
| 3 | 1.80 | 4.20 | 10.10 | 0 |
| 4 | 2.10 | 4.00 | 10.10 | 0 |
| 5 | 2.10 | 4.00 | 9.70 | 0 |
| 6 | 2.07 | 3.87 | 9.50 | 0 |
| 7 | 2.00 | 3.7 | 9.50 | 0 |

Table S2. Parameter details of C5 meta-atoms shown in Fig. 2(h)

| Structure sequence | *a* (mm) | *r* (mm) | *d* (mm) | *θ* (°) |
| --- | --- | --- | --- | --- |
| 1 | 1.70 | 3.60 | 10.38 | 0 |
| 2 | 2.00 | 3.60 | 10.70 | 0 |
| 3 | 1.80 | 3.80 | 10.46 | 0 |
| 4 | 1.70 | 3.70 | 10.62 | 0 |
| 5 | 1.90 | 4.00 | 10.62 | 0 |
| 6 | 1.84 | 4.00 | 10.77 | 0 |
| 7 | 1.70 | 3.60 | 10.38 | 0 |

Table S3. Parameter details of C7 meta-atoms shown in Fig. 2(i)

| Structure sequence | *a* (mm) | *r* (mm) | *d* (mm) | *θ* (°) |
| --- | --- | --- | --- | --- |
| 1 | 2.00 | 3.90 | 10.00 | 0 |
| 2 | 1.82 | 4.00 | 10.42 | 0 |
| 3 | 1.85 | 4.00 | 10.45 | 0 |
| 4 | 1.91 | 4.02 | 10.42 | 0 |
| 5 | 1.95 | 4.10 | 10.10 | 0 |
| 6 | 1.818 | 4.02 | 10.40 | 0 |
| 7 | 2.00 | 3.90 | 10.00 | 0 |

**S6. Details of linear/elliptical-polarization-selective meta-devices**

In this section, we present design details of the linear/elliptical-polarization-selective meta-devices. Figure S7(a) shows the top view of the linear-polarization-selective lens. The prototype is composed of 24×24 meta-atoms, and has dimensions of 264×264 mm^2^. Particularly, the rotation angle of each meta-atom is set as 90° to ensure that each meta-atom is axisymmetric in the *x*-direction. Thus, there is almost no polarization conversion under the illumination of *x*/*y*-polarized waves.

Figure S7(b) presents the top view of the elliptical-polarization-selective lens, which is also composed of 24×24 meta-atoms. To achieve the selective wavefront of a pair of elliptical polarization states, meta-atoms in the array are given uneven rotation angles.


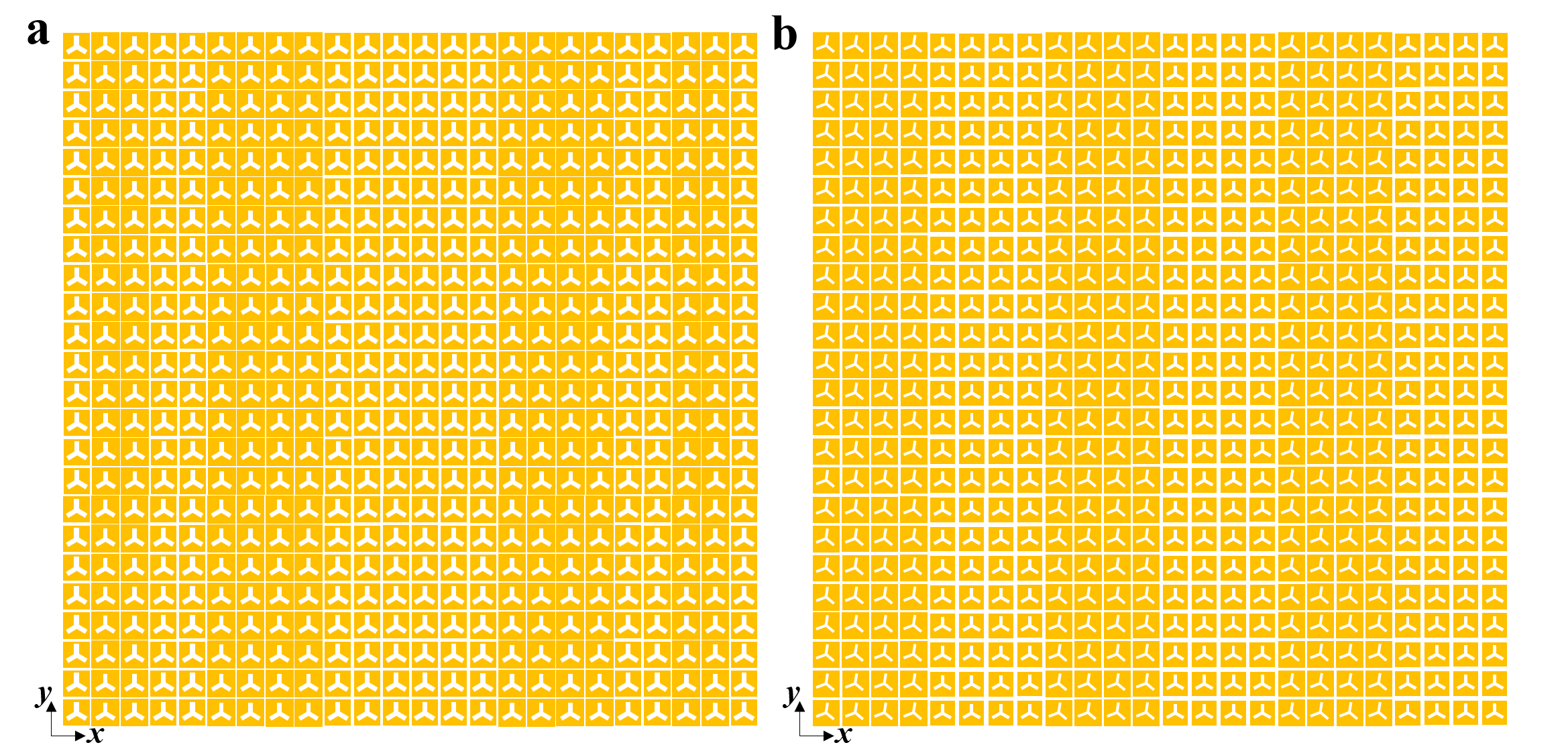


**Figure S7.** Details of (a) the linear-polarization-selective lens and (b) the elliptical-polarization-selective splitter.

**S7. Design of linear/elliptical-polarization-multiplexing meta-devices**

In this section, to more intuitively demonstrate the capability of independent phase modulation for an arbitrary pair of orthogonal polarization states, the polarization-selective devices presented in the previous section is extended to polarization-multiplexed designs. Two representative metasurface examples are provided: one for an orthogonal linear polarization pair and the other for an orthogonal elliptical polarization pair.

Figure S8 presents a linear-polarization-multiplexing meta-reflector. As shown in Fig. S8(a), an anomalous reflection function is implemented under *x*-polarized incidence, with the reflected beam deflected to 15° in the *xoz* plane. Under *y*-polarized illumination, the metasurface functions as a meta-splitter, redirecting the reflected wave into two distinct directions of ±15°. The metallic configuration of the meta-reflector is partially illustrated in Fig. S8(c), which comprises a 24 × 24 array of C3 meta-atoms (for clarity, only 8 meta-atoms are shown along the *y*-direction). A phase sequence of “270°/270°/180°/180°/90°/90°/0°/0°” is applied along the *x*-direction in the reflection channel, resulting in periodic variation of structural parameters every 8 meta-atoms. Along the *y*-direction, adjacent meta-atoms share identical structural parameters. To validate the device performance, full-wave simulations are conducted using a commercial electromagnetic simulation software. Figure S8(d) shows the one-dimensional far-field results in the *xoz* plane. When illuminated by *x*-polarized waves at 12.8 GHz, the reflected beam is deflected to 15°, consistent with the design. In contrast, under *y*-polarized incidence, the reflected wave is split into two directions of −15° and 14°. The intensity difference between the two main beams is approximately 0.05, primarily due to the structural asymmetry of the meta-atoms along the *x*-direction. Overall, the simulation results match the design expectations well and effectively demonstrate the metasurface’s capability to independently tailor the wavefronts of orthogonal linearly polarized waves.


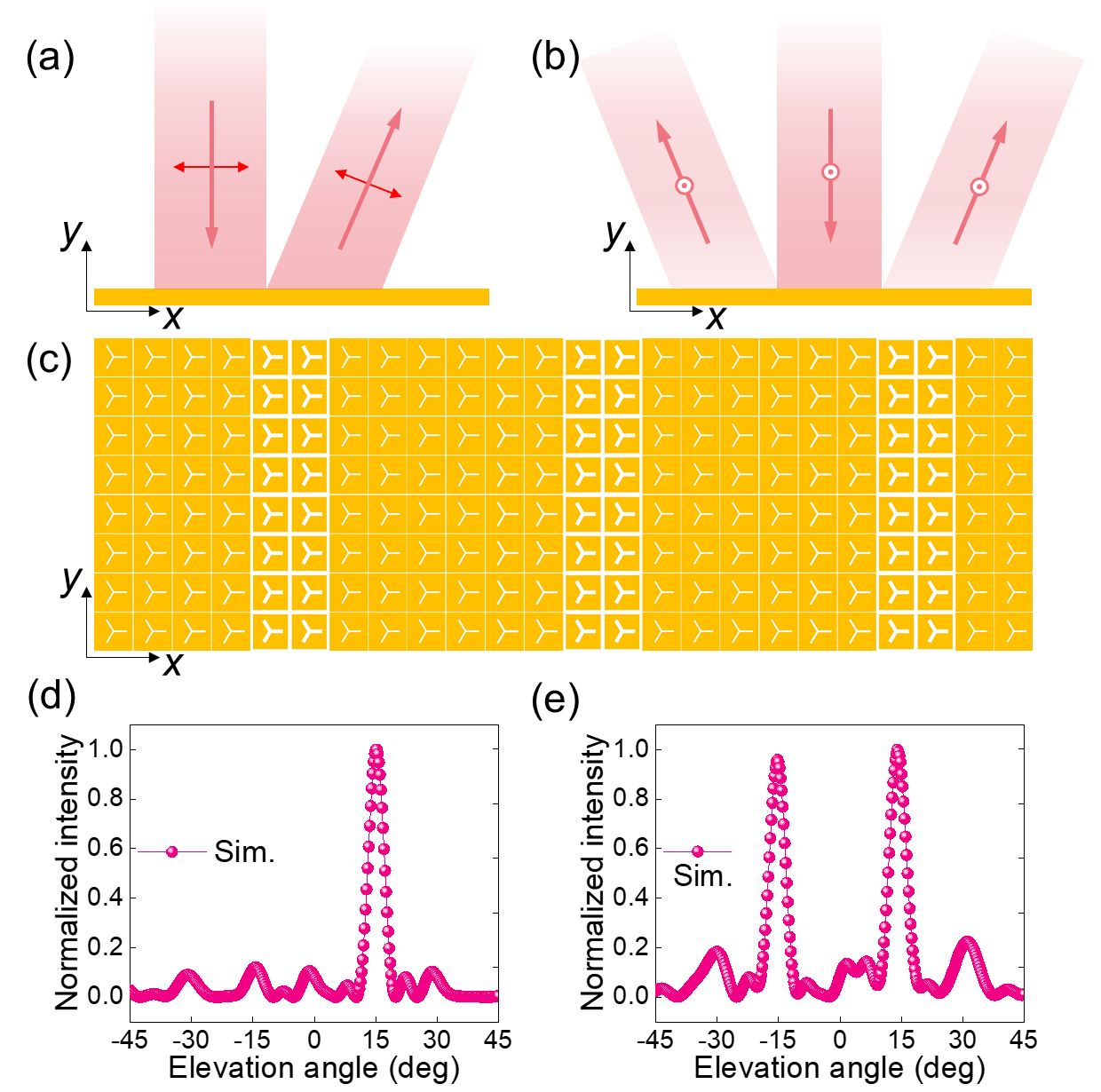


**Figure S8**. Design and performance of linear-polarization-multiplexing meta-devices. Schematic diagram of the C3-based meta-device functioning as (a) a beam deflector at x-polarized incidence and (b) a beam splitter at y-polarized incidence. (c) Details of the meta-device. Far-field simulation performance of the meta-device under (d) x-polarized illumination and (e) y-polarized illumination, respectively.

In order to further demonstrate the phase modulation capability under elliptical polarization, Fig. S9 presents an elliptic-polarization-multiplexing meta-lens. Similar to the configuration in Fig. 4(c) of the main text, a pair of orthogonal elliptical polarization states is selected, defined by *α* = 60° and *χ* = −108°. As shown in Fig. S9(a) and (b), the reflected waves are respectively focused at *z* = 80 mm and *z* = 180 mm under illumination by the right-handed and left-handed elliptical polarization states. The meta-lens shares the same structural scale as the aforementioned linear-polarization-multiplexing meta-reflector and consists of a 24 × 24 array of meta-atoms. Along the *x*-direction, the metasurface is encoded with two distinct focusing phase profiles corresponding to the right-handed and left-handed elliptical polarization channels, enabling distinct phase control. Along the *y*-direction, all meta-atoms within the same column are identical (as schematically shown in Fig. S9(c)). To verify the functionality of the designed meta-lens, full-wave simulations were carried out using a commercial electromagnetic solver. The boundary conditions in the *x*-, *y*-, and *z*-directions were set to “open (add space),” and the metasurface was illuminated with a pair of orthogonal elliptically polarized plane waves. The simulated near-field results are displayed in Fig. S9(d) and (e), showing clear focal spots located at 83 mm and 177 mm, respectively, which are in excellent agreement with the design specifications. These simulation results clearly demonstrate that the proposed metasurface can independently tailor the reflected phase for a pair of elliptical polarization states. This further validates the generality of the polarization multiplexing strategy and highlights its potential for advanced polarization-encoded wavefront engineering.

*
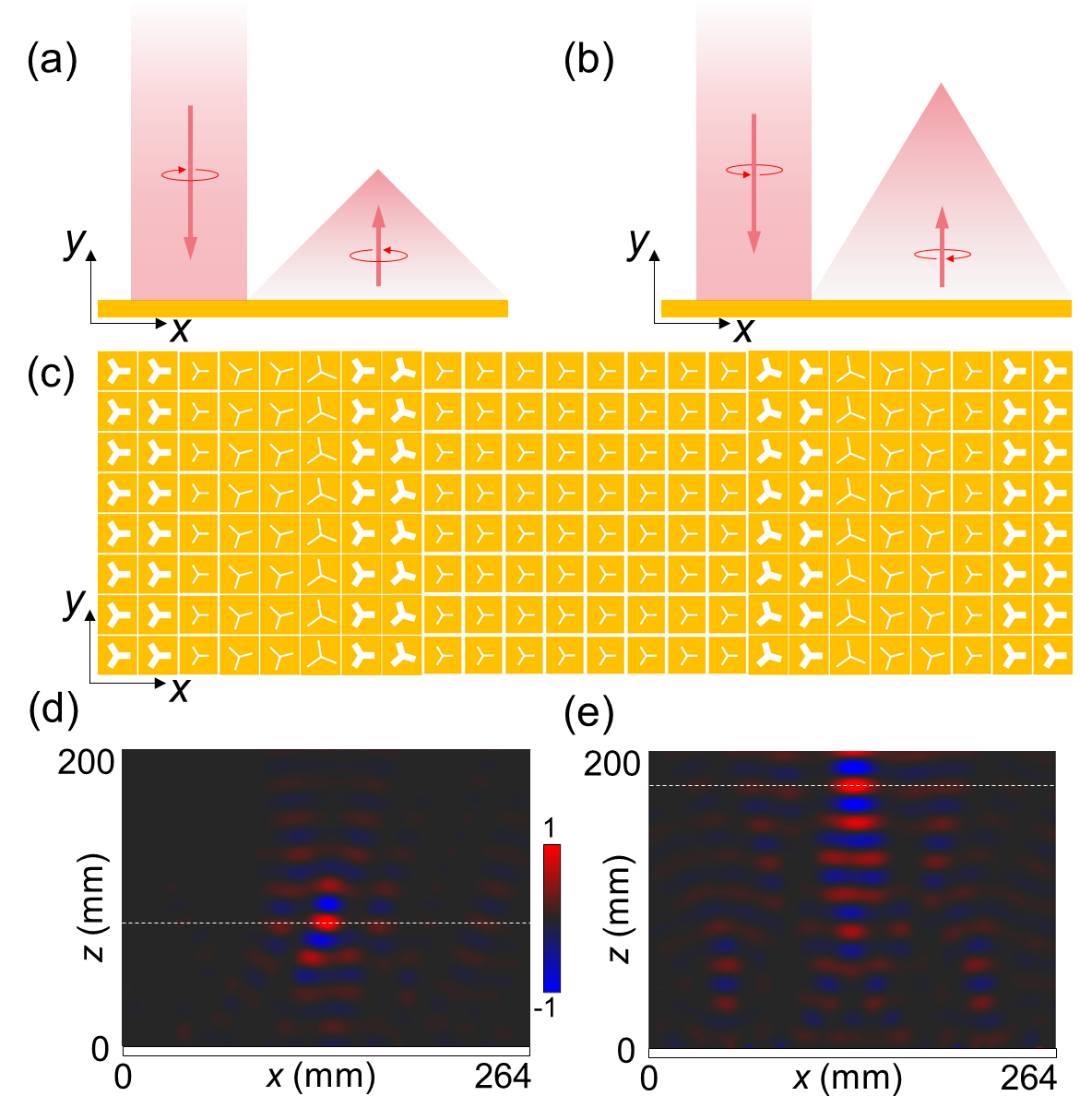
*

**Figure S9**. Design and performance of elliptic-polarization-multiplexing meta-devices. Schematic diagram of the C3-based meta-device functioning as lens with (a) focal length of 100 mm at right-hand elliptic-polarized incidence and (b) focal length of 200 mm at left-hand elliptic-polarized incidence. (c) Details of the meta-device. Near-field simulation performance of the meta-device under (d) right-hand elliptic-polarized illumination and (e) left-hand elliptic-polarized illumination, respectively.

**S8. Experiment setup of the meta-device measurement**

In this section, we provide more details about the measurement of the spin-multiplexing beam meta-deflector and the arbitrary spin-to-orbital angular momentum meta-convertor. As shown in Fig. S10(a), the meta-deflector is measured using a far-field system. Horn antennas, operating in either right-handed circular polarization (RCP) or left-handed circular polarization (LCP) mode, are used to transmit and receive signals at 12.8 GHz. Due to experimental limitations, a planar wave with an oblique incident angle of 5° is used to simulate normal incidence.

Figure S10(b) presents the near-field scanning system used for measuring the arbitrary spin-to-orbital angular momentum meta-convertor. The prototype is placed on a screen of an absorber. A wideband linearly polarized antenna transmits electromagnetic waves at 12.8 GHz to the prototype, and a probe fixed on a 3-D scanning platform detects the reflected field in space. The scanning step is 2 mm, and the detection plane is 500 mm (21*λ*) away from the prototype.





**Figure S10.** Experiment setup of (a) the far-field system and (b) the near-field scanning system.

**S9. C5-based polarization-multiplexing meta-devices**

In this section, two devices based are designed to demonstrate the capability of independent phase manipulation of C5 meta-structures. In Fig. S11, a linear-polarization-selective beam meta-splitter is presented. Under the illumination of *y*-polarized waves, the beam is deflected to directions of ±12°. When *x*-polarized beam is incident, the meta-device can serve as a conventional mirror that reflects the incident beam. Figure S11(b)-(e) shows the details of simulated far-field and near-field results of the meta-device in a pair of orthogonal linear polarization channels. It should be noted that the slight energy difference between the two beams in Fig. S11b is due to the structural asymmetry of the metasurface array in the *x* direction.

Besides, we also design a spin-multiplexing meta-lens is designed to demonstrate independent phase manipulation of a pair of CP waves. The focal lengths in the two CP channels are set to 100 mm (RCP) and 200 mm (LCP), respectively. More details about the meta-lens and its performance are shown in Fig. S12(a)-(e), the independent focusing effect effectively demonstrate the capability of wavefront tailoring of C5 meta-structures.





**Figure S11.** (a) Details of the C5-based linear-polarization-selective meta-splitter. (b)-(c) Simulated far-field intensity of the meta-device under the illumination of *y*-polarized and *x*-polarized waves. (d)-(e) Simulated near-field results corresponding to (b) and (c), respectively.





**Figure S12.** (a) Details of the C5-based circular-polarization-multiplexing meta-lens. (b)-(c) Simulated one-dimensional intensity distribution of the meta-lens under the illumination of RCP and LCP waves. (d)-(e) Simulated reflected near-field distribution corresponding to (b) and (c), respectively.

References

[1] J. P. Balthasar Mueller, N. A. Rubin, R. C. Devlin, B. Groever, F. Capasso, *Phys. Rev. Lett.* **2017**, 118, 113901.

[2] X. Xie, M. Pu, J. Jin, M. Xu, Y. Guo, X. Li, P. Gao, X. Ma, X. Luo, *Phys. Rev. Lett.* **2021**, 126, 183902.
